# Digitalising medical education: sacrificing skills for knowledge?

**DOI:** 10.1080/10872981.2019.1567240

**Published:** 2019-01-20

**Authors:** Lasith Ranasinghe

**Affiliations:** Faculty of Medicine, Department of Medicine, Imperial College London

**Dear Editor,**

The article by Leggett et al. [1] on the use of digital devices for teaching cardiac auscultation shed light on the potential benefit of employing developments in medical technology to educate students. Being a fifth-year medical student, various technology-based educational initiatives have been trialled during my time at medical school, so I feel I can closely relate to the students in this study. As improvements in medical technology come to the forefront, its implementation in medical education seems to be approached with cautious optimism.

Cardiac auscultation is a subtle sensory skill that is often found difficult by many medical students. As such, using technological aids in teaching this topic have been trialled for many years. As early as 1991, Mangione et al. [2]. demonstrated that computer-assisted instruction with graphics and digitised heart sounds was just as effective as seminars at improving the identification of murmurs by students. These early studies, however, were focused on improving the understanding of cardiac auscultation in a classroom setting. The handheld echocardiogram (HHE) and digital stethoscope technique used by Leggett, on the other hand, enable students to engage with real patients whilst following a structured approach to understanding murmurs. The privilege of being able to auscultate a patient and then playback the murmur allows reinforcement of the knowledge gained from the experience. It takes a step away from the commonly heard and frustratingly vague notion that the ability to distinguish heart murmurs ‘*comes with time*’. Furthermore, the inability to standardise the sounds heard by teacher and student in traditional bedside teaching can cause a misinterpretation effect, where the student may alter their recollection of the sounds they heard on auscultation to fit the description of the teacher. This can lead to confusion and a lack of self-confidence in auscultation. Digital stethoscopes mitigate this effect.

Though the use of digital stethoscopes may improve the students’ ability to recognise heart murmurs, it may compromise their practical ability to elicit clear heart sounds from the patient. The reassurance that they can always replay and amplify the sounds later can breed slipshod practice when examining patients. Therefore, employing such technology in teaching sessions may sacrifice practical skills for improved knowledge. Perhaps, a balance must be struck to avoid the encroachment of digitalised learning on the development of practical skills in a clinical setting.

A more moderate approach was demonstrated by Butter et al. [3]. They showed that third-year students trained with a computer tutorial and a cardiac patient simulator demonstrated a significantly higher cardiac auscultation accuracy than untrained fourth-year students when assessing simulated heart sounds and real patients. This shows the benefit of technology-based learning techniques that do not blur the lines between classroom learning and learning in a healthcare setting.

To conclude, though the use of HHE and digital stethoscopes may improve understanding and identification of heart murmurs amongst medical students, it is important to remain aware of the practical skills that may be ceded as a result. Furthermore, an ongoing trend of digitalising medical education is likely to have some impact on the attitudes of students. It is possible that over-reliance on simulation and technology-based teaching may condition students to require ‘spoon-feeding’ and ever more simplification. Therefore, careful attention to students’ attitudes and behaviours should be paid to ensure that their practical acumen and intellectual resilience do not fall as collateral damage to the increasingly digitalised delivery of medical education.
